# Chemodenervation for Oromandibular Dystonia Utilizing Botulinum Toxins

**DOI:** 10.7759/cureus.18425

**Published:** 2021-10-01

**Authors:** Muhammad Atif Ameer, Danish Bhatti

**Affiliations:** 1 Department of Medicine, Punjab Rangers Teaching Hospital, Lahore, PAK; 2 Department of Neurology, University of Nebraska Medical Center, Omaha, USA

**Keywords:** botulinum toxin, focal dystonia, neurology, lingual dystonia, dystonia, oromandibular dystonia, cervical dystonia, botulinum injection

## Abstract

Oromandibular dystonia (OMD) is a chronic focal dystonia that involves the mouth, jaw, and tongue. It may cause repetitive or sustained dystonic movements, which can be very disabling for patients. It is usually a life-long disorder with numerous treatment options that are, most often, partially curative. In our experience, the best modality to treat OMD is botulinum toxin (BoNT) injections, which not only provide long-term relief but also have fewer adverse effects compared to other medications. Although multiple small- and large-scale studies support this fact, there is still a need for evidence from large randomized clinical trials. Jaw-closing dystonia responds very well to BoNT injections compared to other subtypes of OMD. This review discusses in detail the evidence, injection technique, and typical starting doses for botulinum injection.

## Introduction and background

Primary oromandibular dystonia (OMD) is a type of dystonia that involves the mouth, jaw, and tongue [1]. OMD is usually seen in conjunction with numerous neurological and non-neurological diseases [2,3]. It is an uncommon condition with a prevalence of approximately 70 per million with a female predominance [4]. Several oral pharmacological agents (benzodiazepine, zolpidem, tetrabenazine, baclofen, dopaminergic agents) have been tried. However, their use is limited due to high cost, inadequate response, and side effect profile, including suicidal ideation and depression [5,6].

OMD is one of the most disabling dystonia, and patients affected with this condition are often stigmatized by visual deformity and social embarrassment, which leads to social withdrawal and numerous psychiatric conditions [7]. In clinical practice, botulinum toxin (BoNT) injection is considered to be the most effective treatment for OMD. Several large- and small-scale studies support the fact that BoNT provides excellent benefits. Proper technique, background knowledge of the oral anatomy, and serotype used provide remarkable results with only minor self-limiting adverse effects [8,9].

A comprehensive search of databases and search engines, including PubMed/Medline and Google Scholar, was performed using appropriate keywords, including oromandibular dystonia, BoNT, botulinum toxin, and chemodenervation. Articles with the potential of contributing to the chemodenervation of OMD were included in the review. Here, the information gathered from the literature search is integrated with clinical expertise and presented as a comprehensive guideline to assist the clinical practice involving chemodenervation of OMD utilizing BoNT.

## Review

Types of oromandibular dystonia

OMD is a focal dystonia that affects lower facial muscles and is classified as jaw-closing dystonia, jaw-opening dystonia, jaw-deviating dystonia, lingual dystonia, perioral dystonia, or a combination of these (Table 1) [10].

**Table 1:** Muscles involved in different types of oromandibular dystonias.

Types	Muscle(s) involved
Jaw-opening dystonia	Lateral pterygoid, digastric
Jaw-closing dystonia	Masseter, temporalis
Jaw-deviating and protrusion dystonia	Lateral pterygoid, medial pterygoid
Lingual dystonia	Genioglossus
Perioral dystonia	Orbicularis oris, platysma, zygomaticus, risorius levator nasii labii, levator anguli oris, depressor anguli oris, mentalis

Facial dystonia may involve both the upper and lower face and presents with perioral dystonia. Similarly, the contribution of blepharospasm (involuntary closure of eyes) and OMD together is known as Meige syndrome [11]. Currently, there are no standard diagnostic criteria for OMD, and the diagnosis is mainly clinical. Isolated OMD is uncommon, accounting for only 5% of all forms of dystonia. Among the different classifications of OMD, jaw-closing dystonia is the most responsive to botulinum injections.

These forced movements can cause intraoral soft tissue trauma, including cheek and tongue biting, subluxation and mandible deviation, temporomandibular joint dysfunction, arthritis, and bone resorption. Clinically, these inappropriate movements can present as dysphonia, dysphagia, and difficulty in mastication [12]. Patients with OMD frequently present to dentists for dentures and are frequently under-recognized [13,14].

The current state of evidence

According to a class I clinical practice guideline, currently, there is a level C recommendation for Abo-BoNT and Ona-BoNT, whereas a level U recommendation for Inco-BoNT A and Rima-BoNT B, given the lack of randomized control studies [15]. One of the earliest reports of the use of BoNT in 1989 used Oculinum (older formulations of BOTOX, Onabotulinumtoxin A) in divided doses of 10-40 units per muscle using electromyography (EMG) guidance and showed on average 47% improvement in OMD patients [16]. A study of jaw-closing dystonia used botulinum toxin type A (BTX-A) with a mean dose of 54.2 U (±15.2 U) for the masseters and 28.6 U (±16.7 U) for the submentalis. Patients showed remarkable results with a global mean effect of 3.1 U (±1 U) on a scale of 0-4 (where 4 = complete resolution of dystonia) [17].

A review of 421 patients with dystonia identified 17 cases (age ranging from 19 to 77 years) with lingual dystonia (tongue protrusion) with various etiologies, including tardive dystonia. The majority of the patients had jaw dystonia. Of the 17 cases, nine were treated with BTX-A in the genioglossus muscle. Of these nine patients, two had no response, one had a mild response for a few weeks (30 units on each genioglossus), one had a moderate response, and five patients had an excellent response. This amounts to a 66.3% excellent response rate out of the total toxin injection sessions and a 15.7% moderate response. There was only one case of severe dysphagia requiring percutaneous gastrostomy tube placement [18].

A meta-analysis published in 2018 reviewed the published literature and selected nine studies of BTX-A for inclusion after excluding several cases reports and case series as well as some small studies which did not report safety outcomes [14]. A total of 387 patients with isolated OMD were selected for the meta-analysis after excluding patients injected for other indications concomitantly. The results showed a pooled average reduction of 39% of dystonic movements in the treated group compared to the control group. Nevertheless, there was significant heterogeneity of 86.7% due to between-study variance and differences. Although the study showed no significant publication bias, there was no overestimation of the effect of intervention compared to control. Overall, a 27% complication rate was noted, with the most common side effect being dysphagia. Other side effects included jaw weakness, weak smile, nasal regurgitation, jaw tremors, mouth dryness, dysarthria, lip numbness, and pain at the injection site [14].

Rima-BoNT type B toxin has not been thoroughly studied in OMD and is typically not the first treatment choice. In a small open-label study of four patients, two patients with OMD (segmental dystonias) who became resistant to BTX-A were treated successfully with BTX-B (Rima-BoNT), suggesting that BTX-B injections are a safe and effective option for the management of OMD patients who become secondary nonresponders to BTX-A [19]. In a prospective observational study of 477 patients with focal dystonia, including 62 patients with OMD, patients were injected at the anterior temporalis, submental complex (digastric), pterygoid, and masseter. They received 407 injections in total, with the dose peaking at 50 units of Ona-BoNT. Results were determined by patients on a scale of 0 (no change) to 4 (marked change). Patients reported mild-to-moderate benefits at the peak of the treatment [11].

Injection technique

Here, we discuss the most commonly injected muscles in different types of OMD (Table 2).

**Table 2:** Most commonly injected muscles in oromandibular dystonias.

Dystonia classification	Commonly injected muscle
Jaw-closing dystonia	Masseters, temporalis, medial pterygoids
Jaw-opening dystonia	Submentalis, anterior belly of digastric, lateral pterygoids, platysma
Jaw deviation	Lateral pterygoid (contralateral) and temporalis (ipsilateral)
Lingual dystonia	Genioglossus, hyoglossus

Jaw-Closing Dystonia

The masseter is ideally injected 1 cm anterior to the posterior border of the ramus. The starting dose for masseter is 15-25 units of Ona-BoNT and Inco-BoNT, whereas 60-100 units of Abo-BoNT [20-22]. The temporalis is ideally approached perpendicularly as high as possible due to the fan-shaped anatomy of the muscle. For temporalis, the starting dose is 10-20 units for Ona-BoNT and Inco-BoNT and 60-80 units for Abo-BoNT [20-23].

Jaw-Opening Dystonia

For the lateral pterygoid external approach, the needle is ideally inserted approximately 35 mm away from the external auditory meatus and inferiorly 10 mm from the zygomatic arch. The needle is kept upward at 15 degrees to enter the external pterygoid inferior head, utilizing EMG. The usual doses are 10-30 units of Ona-BoNT and Inco-BoNT or 30-60 units of Abo-BoNT. Several experts prefer the intraoral approach compared to the extraoral approach [12,20,21]. Digastric is ideally injected with the patient seated supine with the head tilted backward, and the needle is placed 1 cm from the middle of the neck and 1 cm behind the jaw bone [18,24]. The typically used dose for digastric is 5-10 units of Ona-BoNT and Inco-BoNT and 10-30 units of Abo-BoNT on each side [24,25].

Lingual Dystonia

Genioglossus forms the floor of the tongue and can be approached both intraorally and extraorally. The extraoral technique is the same as digastric, while the needle usually pierces the digastric first and then enters the genioglossus [18]. The usual dose for lingual dystonia is 10 units of Ona-BoNT or Inco-BoNT and 20-30 units of Abo-BoNT [15].

Adverse effects

Patients usually experience myalgias, pain at the injection site (bruising), dizziness, skin rash, and dry mouth [26,27]. One of the most feared complications after BoNT injection is dysphagia, which particularly occurs while injecting lingual dystonia and is by far the most challenging dystonia to treat. Dysarthria, dysphonia, and difficulty in chewing have also been described in a minority of the patients [28,29]. With experienced professionals, a majority of the patients experience no adverse effects, while those who suffer adverse effects are usually minor and self-resolving [30].

## Conclusions

OMD affects masticatory, lower facial, and lingual muscles. The involvement of these muscles produces painful spasms and abnormal movements of the face that interfere with everyday life activities and may result in social embarrassment. In our experience and available literature, treatment with botulinum is the most effective compared to other modalities. BoNT injection requires careful dosing, familiarity with oromandibular anatomy, and injection technique for better results. However, there is still a need for extensive research to understand the exact pathology of dystonia and the use of BoNT. In the future, controlled studies should provide better evidence for the most appropriate site selection method for BoNT injection.
